# Comparison of attitudes toward the medical student-led community health education service to support chronic disease self-management among students, faculty and patients

**DOI:** 10.1186/s12909-023-04008-7

**Published:** 2023-01-11

**Authors:** Shi Wang, Dan Yan, Xianmin Hu, Juan Liu, Dan Liu, Jun Wang

**Affiliations:** Medical College, Wuhan University of Science and Technology, Wuhan, China

**Keywords:** Community service learning, Medical student-led community health education service, Chronic disease self-management, Stakeholders, Medical students, Faculty, Patients, Attitudes, Comparison

## Abstract

**Background:**

Shortage of health professionals is one of the most important barriers for community health centers to provide quality primary care for chronic disease patients especially after the outbreak of COVID-19. Under such condition, medical students have been well-accepted as a force multiplier for community-based health service. Community service learning (CSL) based on medical student-led community health education service to support chronic disease self-management might be a valuable interactive learning tool in medical education. This study compared the attitudes toward medical student-led community health education service to support chronic disease self-management among three stakeholder roles in CSL, including medical students, faculty and patients.

**Methods:**

This cross-sectional comparative survey was conducted using a self-developed questionnaire among the convenience samples of undergraduate students and faculty members from the Medical College, Wuhan University of Science and Technology, as well as patient volunteers with chronic diseases recruited from a free on-site clinic offered by a community health center. Attitudes toward medical student-led community health education service to support chronic disease self-management were compared among students, faculty and patients.

**Results:**

A total of 515 valid questionnaires were obtained (342 were collected from medical students, 54 from faculty respondents, and 119 from patients). Overall positive attitudes toward medical student-led community health education service to support chronic disease self-management were positive. Among the three stakeholder roles, faculty and patients were more supportive of the current inadequate level of primary care provision within the community. However, patient respondents showed more negative attitudes towards using resources in higher medical education system to provide support for primary care practice, and participating in the medical student-led community health education service to support chronic disease self-management, and were most skeptical about the medical students’ competency in supporting chronic disease self-management with their professional knowledge and skills. The educational value of CSL for medical undergraduates and the role of faculty instructors were most appreciated by faculty respondents. Additionally, > 62 years old and > 2 kinds of chronic diseases per patient exhibited significant correlations with positive patients’ attitudes.

**Conclusions:**

Medical students, faculty and patients had overall positive attitudes towards CSL based on medical student-led community health education service to support chronic disease self-management. However, more should be done to create higher expectations and enthusiasm of patients about CSL.

## Background

So far, the global burden of non-communicable chronic diseases represented by hypertension, coronary heart disease, stroke and diabetes, etc. has been a significant public health concern as a result from accelerated ageing of the population around the world [1–3]. Uncontrolled chronic diseases are closely associated with diverse severe co-morbidities and adverse outcomes. According to the World Health Organization estimates, in 2016, approximately 40.5 million people died of non-communicable chronic diseases, which accounted for 71% of the total worldwide deaths within year [4]. Long-term and comprehensive health-care interventions associated with chronic diseases including population-based prevention, primary and secondary prevention for high-risk individuals via multi-drug therapy, co-morbidity management, and treatment of acute cases, etc. caused heavy financial burdens for patients and nations, which posed a formidable challenge especially for resource-limited developing countries [5–7]. In China, it was estimated that nearly 75% of the elderly population aged 60 and over had at least one kind of chronic disease [1, 6], and chronic diseases contributed to about 70% of the total national health expenditures [5]. So far, a multi-tiered healthcare system including tertiary hospitals, secondary hospitals, and primary care facilities with an emphasis on the role of primary care facilities (e.g. community health centers) has been globally accepted as a cost-effective and sustainable way for managing chronic diseases [2, 3, 5, 8, 9]. In China, community-based primary care is currently the first level of contact for chronic disease patients into the health system, and often acts as the principal avenue for chronic disease management through the provision of community-focused, affordable, comprehensive, family-oriented, long-term and continuous care closer to home [3, 5].

Among the comprehensive management strategies for chronic diseases, the effective daily self-management support has been accepted as one of essential components for high-quality chronic disease management [10]. Chronic disease self-management approaches included a package of behavioural interventions addressing the medical, physical, emotional, social and related challenges posed by the chronic diseases, aiming to improve health-related attitude and quality of life, as well as to reduce chronic disease-related complications and mortality. Successful self-management experience on health behaviors and lifestyle intervention such as diet, routinized physical activity, medication, and attendance at follow-up appointments has been demonstrated to be consistent with better health outcomes [11]. Accordingly, chronic disease self-management education via effective health communication among the patients and the health-care providers is necessary to support and improve patients’ self-efficacy, knowledge, self-care skills, and adherence to recommended self-care behaviors [12]. For patients with chronic diseases, the community-based primary care system is the frontline responsible for health education service to support chronic disease self-management [9]. Under the direction of community health educators, patients can ensure their treatment adherence, make lifestyle adjustments, make decisions on management options and actions, identify possible challenges and solve them as early as possible [9]. According to the Basic Public Health Services launched by Chinese central government in 2009, health education as one of the most important programs has been requested to be freely provided for all community residents in response to their needs by community health centers [12].

Despite the increasing recognition of the importance of community-based primary care in chronic disease management especially in health education service, studies conducted in different countries point out that the lack of health providers is one of the most important barriers for community health centers to provide quality primary care for chronic disease patients [13–16]. Similar to most countries in the world, China is currently facing the transformation from the traditional hospital-centric healthcare system to the community-based primary healthcare model. Accordingly, the shortage of community health professionals has been considered as a key issue hindering the development of primary care in China [16]. In fact, under the current circumstance, community health education is often performed by community nurses as part-time workers [5, 6]. It appears to be difficult to attract enough qualified health educators in community health institutions within a short period of time, due to the constraints of the current working conditions, income and training cycle, etc.

Based on their vibrancy, acquired professional knowledge and skills, as well as their awareness of local cultural customs, medical students have been well-accepted as a force multiplier for the community-based health service [17–20]. For example, medical students were involved in a student-led community-based prevention program for falls, which has been demonstrated to potentially enhance the sustainability of the program and reduce program costs [17]. Our team previously recruited undergraduate students from medical and other health professions to participate in household visits for community residents suffering from diabetes aiming to increase diabetes self-management education [18]. Through a team-based model, medical students can channel their energy and passions to elevate the community health in any way they can [18, 19]. Especially as the current COVID-19 pandemic has challenged and, in many cases, exceeded the capacity of healthcare systems worldwide, more and more medical students have engaged in patient care–related community services, and contributed greatly to improve community public health [19, 20]. Student-led health clinics offer valuable and potential cost-saving opportunities for meeting the health care needs of residents especially in underserved communities while also for student professional learning [21, 22].

As an interactive learning tool in experiential education engaging students in human-centered service-learning activities to address community needs as well as develop students’ professional value and knowledge, the community service learning (CSL) has achieved broad acceptance among medical schools and educators [18, 23–28]. So far, the positive educational effects of CSL have been demonstrated at all levels of learning for the cognitive, affective, and psychomotor domains [27]. Through providing an authentic sociocultural context for medical professional education, CSL allows medical students to gain exposure to primary care and social justice, flexibly apply classroom knowledge in real life, and strengthen their learning, professionalism, communication and problem-solving skills, civic and social responsibility as well as sense of community [25–28]. Especially for health professions previously being therapy-oriented, there is more emphasis now on prevention and primary care via improving life styles and eliminating risk factors having adverse effects on public health. Accordingly, in order to serve the educational needs of modern medicine in the twenty-first Century, integrating community service into student projects provides opportunities for medical students to gain competency in public health, preventive practice and social service through the learning experience of actually delivering health care to community residents in need [27, 28]. As for medical students, personal experience of contact with real patients is certainly an integral component of their professional learning. Traditionally, most medical student contact with patients occurs during their clinical placements in teaching hospitals. CSL offers students especially at preclinical stage the additional benefits such as a focus on person rather than disease, and a clear presentation of the influence of social factors on health care [29]. However, the above pedagogic advantages of CSL programmes were considered and addressed mainly in terms of student dimensions. Attitudes from other stakeholders (e.g. faculty members as instructors, patients as beneficiaries) regarding their involvement in CSL as stakeholders have gone largely unexplored [27]. Therefore, the present study aimed to determine whether a gap exists among the attitudes of the involved stakeholders, including students, faculty and patients, towards the CSL-based medical student-led community health education service to support chronic disease self-management. The findings would better support the future CSL activities in medical education as well as the community health service practices for patients with chronic diseases.

## Methods

### Study design

This cross-sectional survey assessed and compared the attitudes about medical student-led community health education service to support chronic disease self-management among three different stakeholder populations, including students, faculty and patients, using a self-developed questionnaire.

The survey among student and faculty respondents was conducted in the Medical College, Wuhan University of Science and Technology, over a period of 3 months from May to July, 2022. As a typical provincial comprehensive university located in the central China, the sampling university enrolls medical students from all the country. The Medical College of the university has a history of more than 60 years, and has been certified by the Chinese Ministry of Education clinical professional certification working committee. The curriculum design, patient casemix, and staff profile, etc. in the Medical College are in accord with the national uniform standards for undergraduate medical education, thus similar to those at other medical schools in China. Currently, there are approximately 1200 enrolled undergraduate medical students and 200 faculty members in the Medical College. On each Monday during the survey period, the research team members set a booth at the college hall where undergraduate students and teaching faculty members from the Medical College could be randomly approached and face-to-face invited to complete the online anonymous questionnaire. The sample size of students was calculated using the online Raosoft sample size calculator [30] based on a margin of error of 5%, a confidence interval of 95%, and a response rate of 50%, thus the required sample size of students was 292. Accordingly, this study recruited a convenience sample of 348 student participants, which accounted for about a quarter of the enrolled undergraduate medical students in the Medical College. The proportion to population size sampling technique was used to calculate the number of faculty members to be selected. The eventually collected data were from 54 faculty participants, representing a quarter of the total number of faculty members in the Medical College.

Patient volunteers with chronic diseases were recruited from a free on-site clinic offered by a community health center on July 3, 2022. According to medical records in the community health center, all community residents diagnosed with non-communicable chronic diseases including hypertension, type 2 diabetes, hyperlipemia, cardiovascular diseases, stroke, cancer, neurological disorders, chronic kidney or liver diseases, chronic arthritis, chronic endocrine or respiratory diseases, etc. were invited by phone call made by the community nurses to participate in this free on-site clinic. The healthcare professionals in the free on-site clinic were not the research team members. After the clinic visit, the patients were invited to voluntarily complete the anonymous survey. The patients were informed that, regardless of whether they participated in the survey or not, they would receive usual healthcare at the community health center. Considering that most chronic disease patients were elder, patient respondents provided their responses to the paper questionnaire marked by the researchers. The chronic disease patients with aphasia, dementia or any psychiatric diagnosis were eliminated from the survey. The study protocol was approved by the Ethics Committee of Medicine College, Wuhan University of Science and Technology.

### Questionnaire

The participants were informed about the aims of this survey and the idea of CSL as an explanatory letter at the beginning of the questionnaire. The initial questionnaire items were developed by the research team based on existing literature about CSL [18, 23–29]. The clarity, content validity, relevance, and conciseness of the questionnaire items were appraised by 2 specialists in medical education external to the research team. A pilot test was performed on a convenience sample including 10 patients with chronic diseases, 20 undergraduate students and 5 teachers in the studied medical college who were not included in the final survey. The Cronbach’s α value obtained from the pilot test was 0.765. After a minor modification in the wording and contents based on feedback from 2 specialists and 35 respondents mentioned above, the formal survey questionnaire was obtained.

The final questionnaire was in Chinese and included three parts. The first part included elements designed to collect the respondents’ demographic data. Concretely speaking, the faculty respondents were asked about their gender, profession, duration of engagement in medical education, and whether they had previously introduced case-teaching in their own teaching classes. Students were asked about their gender and grade levels. Patients were asked about their gender, age, chronic disease(s) that they had, as well as whether they had previously received community health education service provided by professionals from the community health center to support chronic disease self-management. In the second part, 8 questions (Part II-Q1–8) were designed to capture the attitudes of students, faculty members and patients toward the CSL-based medical student-led community health education service to support chronic disease self-management, the answers of which were recorded using a five-point Likert scale ranging from “strongly disagree” to “strongly agree”. An additional single-choice question in the second part was about the students’, teachers’ and patients’ preferred mode of medical student-led community health education service (Part II-Q9). The last part requested responses regarding the students’ and teachers’ attitudes on the possible educational benefits of medical student-led community health education service to support chronic disease self-management, as well as their self-confidence in participating in the related CSL programs.

### Statistical analysis

The collected data were entered into SPSS 25.0 for analysis. Results were shown as numbers (percentages) for categorical variables and mean ± standard deviation (SD) for quantitative variables. The categorical data were statistically analyzed using the χ2 test. The Independent *t *test was applied to compare quantitative data between two groups. One-way analysis of variance (ANOVA) with post hoc Tukey’s honestly significant difference analysis was conducted for multiple comparisons among three or more groups. In order to determining which patients would be most amenable to accept this intervention, multiple logistic regression analyses were performed to assess independent associations between hypothesized predictors (gender, age, number of chronic disease(s) that patients had, as well as their experience of receiving community health education service to support chronic disease self-management) and each attitude statement. *p* < 0.05 was deemed statistically significant.

## Results

### Descriptive statistics

By the end of the survey period, 348 (95.1%) of 366 randomly approached medical students and 54 (98.2%) of 55 contacted faculty members responded to the survey, yielding 342 and 54 completed questionnaires from students and teachers (overall effective response rate: 93.4 and 98.2%), respectively. Among a total of 155 patients visiting the on-site clinic offered by the community health center, 119 completed the questionnaires with a response rate of 76.8%. Together, a total of 515 valid questionnaires were obtained for analysis.

Respondents’ characteristics were reported in Table 1. The gender distribution of respondents was approximately balanced. Medical student respondents were from all grades of 5-year medical undergraduate program. Half of teacher respondents had worked as faculty members for 11 to 20 years. A vast majority (90.7%) of faculty respondents had previously introduced case-teaching in their own teaching classes. The age range of the responding patients with chronic disease was between 45 and 89 years, with an average age of 62.41 ± 10.20 years. Among them, there were 57 patients aged over 62 years and 62 ones aged 62 years and below. Of the 119 patient respondents, only half (51.3%) stated that they had ever received community health education service provided by professionals from the the community health center to support their chronic disease self-management.

**Table 1:** Demographic data of the respondents

Characteristics	Medical students (*n* = 342)	Faculty members (*n* = 54)	Patients (*n* = 119)
**Gender,** n(%)			
•Male	148(43)	26(48)	62(52)
•Female	194(57)	28(52)	57(48)
**Age**, Mean ± SD	–	–	62.41 ± 10.20
**Grade,** n(%)			
•First-year	80(23)	–	–
•Second-year	106(31)	–	–
•Third-year	52(15)	–	–
•Fourth-year	54(16)	–	–
•Fifth-year	50(15)	–	–
**Duration of engagement in medical education**, n(%)			
•1–5 years	–	7(13)	–
•6–10 years	–	7(13)	–
•11–20 years	–	27(50)	–
•21–30 years	–	7(13)	–
• > 30 years	–	6(11)	–
**Profession**, n(%)			
•Preclinical medicine	–	20(38)	–
•Clinical medicine	–	17(31)	–
•Preventive medicine	–	17(31)	–
**Whether the responding faculty members had previously introduced case-teaching in their own teaching classes?** n(%)			
•Yes	–	49(91)	–
•No	–	5(9)	–
**Number of chronic diseases per patient**, Mean ± SD	–	–	2.55 ± 1.16
**Whether the responding patients had previously received community health education service provided by professionals from the the community health center to support chronic disease self-management?** n(%)			
•Yes	–	–	61(51)
•No	–	–	58(49)

-: Not applicable

### Attitude comparison among students, faculty and patients

All respondents were required to rate the statements about the CSL-based medical student-led community health education service to support chronic disease self-management using a five-point Likert scale. As shown in Table 2, the student, faculty and patient respondents only showed their consistent agreement with one statement that “Compared with the qualified physicians, the medical students are more vibrant, enthusiastic, patient and available to provide individualized healthcare service**”** (Part II-Q6, *p* > 0.05)**.** Compared to the medical students, faculty members and patients were significantly more supportive of the current inadequate level of primary care provision within the community (Part II-Q1, *p* < 0.001). Moreover, most of patients (69.8 and 63.9%, respectively) agreed or strongly agreed that “Manpower, intelligence and professional knowledge in higher medical education system can provide support for primary care practice” (Part II-Q2) and “I am very pleased to participate in the medical student-led community health education service to support chronic disease self-management” (Part II-Q3). About half (49.6%) patient respondents agreed or strongly agreed the statement that “According to my estimate, community patients with chronic diseases are looking forward to the medical student-led community health education service to support chronic disease self-management” (Part II-Q5). Nevertheless, compared to students and teachers, the patient respondents showed more negative attitudes towards using resources in higher medical education system to provide support for primary care practice (Part II-Q2), and participating in the medical student-led community health education service to support chronic disease self-management (Part II-Q3 and 5) (*p* < 0.01). The value of CSL for medical undergraduates (Part II-Q4) and the role of faculty instructors (Part II-Q8) in CSL were most appreciated by the faculty respondents (*p* < 0.001). In addition, the mean attitude score of respondents towards the statement of “Medical students have acquired enough professional knowledge and skills to support chronic disease self-management.” (Part II-Q7) was 3.63 ± 0.99 out of 5. Among the three stakeholder roles, the patient respondents were most skeptical about the medical students’ competency in supporting chronic disease self-management with their professional knowledge and skills (*p* = 0.015), with only 46.2% responding patients who agreed or strongly agreed the survey statement of Part II-Q7.

**Table 2:** Attitudes toward the medical student-led community health education service to support chronic disease self-management (Mean ± SD)

Survey Question/Statement	Medical students (*n* = 342)	Faculty members (*n* = 54)	Patients (*n* = 119)	*p* value*
Part II-Q1: The current level of primary care provision within the community is inadequate to address the healthcare needs of patients with chronic diseases**.**	4.01 ± 0.77	4.28 ± 0.63	4.29 ± 0.79	< 0.001
Part II-Q2: Manpower, intelligence and professional knowledge in higher medical education system can provide support for primary care practice.	4.08 ± 0.73	4.26 ± 0.56	3.88 ± 0.72	0.0031
Part II-Q3: I am very pleased to participate in the medical student-led community health education service to support chronic disease self-management.	4.33 ± 0.70	4.35 ± 0.68	3.73 ± 0.94	< 0.001
Part II-Q4: As for the medical undergraduates, CSL is a valuable learning method.	4.21 ± 0.73	4.63 ± 0.49	4.35 ± 0.66	< 0.001
Part II-Q5: According to my estimate, community patients with chronic diseases are looking forward to the medical student-led community health education service to support chronic disease self-management**.**	4.10 ± 0.81	4.06 ± 0.71	3.46 ± 0.70	< 0.001
Part II-Q6: Compared with the qualified physicians, the medical students are more vibrant, enthusiastic, patient and available to provide individualized healthcare service**.**	4.31 ± 0.78	4.04 ± 0.89	4.28 ± 0.68	0.120
Part II-Q7: Medical students have acquired enough professional knowledge and skills to support chronic disease self-management.	3.66 ± 1.02	3.85 ± 0.86	3.42 ± 0.93	0.015
Part II-Q8: Having competent faculty instructors to guide and supervise medical students is crucial for the medical student-led community health education service to support chronic disease self-management.	4.41 ± 0.66	4.78 ± 0.42	4.47 ± 0.61	< 0.001

* One-way ANOVA with post hoc Tukey’s HSD

**Fig. 1:**
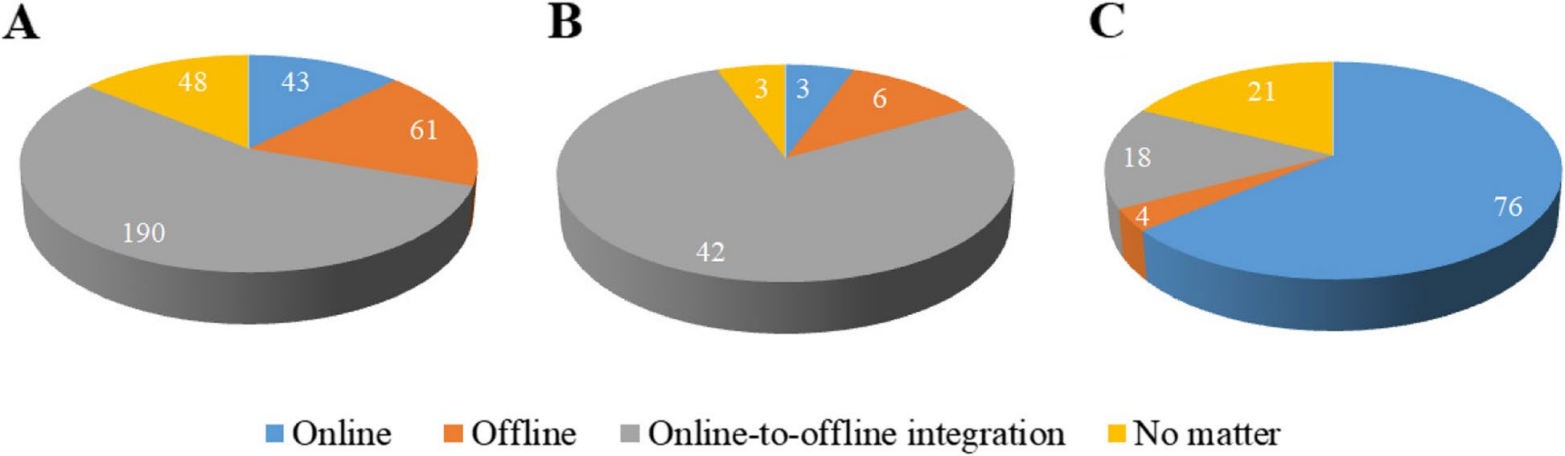
The preferred mode of medical student-led community health education service to support chronic disease self-management among medical students (**A**), faculty members (**B**) and patients (**C**)

Furthermore, we explored the preferred mode of medical student-led community health education service to support chronic disease self-management among students, teachers and patients. As shown in Fig. 1, there was a significant statistic difference in the mode preference (χ2 = 164.38, *p* < 0.001). The most preferred mode for medical students and faculty was the online-to-offline integration, which was supported by 55.6% of student respondents and 77.8% responding teachers, respectively. However, most (63.9%) patients with chronic disease reported that they wanted to participate in the online community health education service led by medical students.

### Predictive factors affecting the patients’ attitudes

In order to identify the patient candidates for future CSL programs or activities based on medical student-led community health education service to support chronic disease self-management, we conducted multiple logistic regression analyses with patients’ attitudes (Part II-Q1–8) as the explanatory variables to elucidate the background factors for positive attitudes of patients. The hypothesized predictors included the patients’ gender, age, number of chronic disease(s) that they had, as well as their experience of receiving community health education service to support chronic disease self-management. As shown in Table 3, compared to the younger respondents, patients aged over 62 year appeared to have significantly more positive attitudes toward the statements of “I am very pleased to participate in the medical student-led community health education service to support chronic disease self-management”(Part II-Q3), “According to my estimate, community patients with chronic diseases are looking forward to the medical student-led community health education service to support chronic disease self-management.” (Part II-Q5) and “Medical students have acquired enough professional knowledge and skills to support chronic disease self-management.”(Part II-Q7) (*p* < 0.01). Patients with more than two kinds of chronic diseases tend to more agreeable with the patients’ expectations for the medical student-led community health education service to support chronic disease self-management (Part II-Q5) as well as the medical students’ competency (Part II-Q7) (*p* < 0.05; *p* < 0.01) than those having two or less kinds of chronic diseases. Moreover, those having experience of receiving community health education service to support chronic disease self-management felt more certain about only one statement “The current level of primary care provision within the community is inadequate to address the healthcare needs of patients with chronic diseases”(Part II-Q1) (*p* < 0.01). However, patients’ attitudes had no statistically significant correlation with gender.

**Table 3:** Multiple logistic regression analysis with patients’ attitudes toward the medical student-led community health education service to support chronic disease self-management

Characteristics	Odds ratio (95% confidence interval)							
	Part II-Q1	Part II-Q2	Part II-Q3	Part II-Q4	Part II-Q5	Part II-Q6	Part II-Q7	Part II-Q8
**Gender**								
•Male(Reference)	1.00	1.00	1.00	1.00	1.00	1.00	1.00	1.00
•Female	1.33(0.65,2.73)	1.11(0.54,2.28)	0.93(0.46,1.88)	0.52(0.25,1.09)	1.11(0.52,2.38)	1.80(0.85,3.80)	0.51(0.25,1.06)	1.05(0.50,2.23)
**Age**								
≤ 62 years (Reference)	1.00	1.00	1.00	1.00	1.00	1.00	1.00	1.00
> 62 years	1.36(0.66,2.80)	1.44(0.70,2.97)	4.36(2.05,9.30)^b^	1.73(0.83,3.62)	7.35(3.21,16.80)^b^	2.45(1.15,5.22)	3.70(1.74,7.85)^b^	0.98(0.46,2.09)
**Number of chronic diseases per patient**								
≤ 2 (Reference)	1.00	1.00	1.00	1.00	1.00	1.00	1.00	1.00
> 2	0.82(0.39,1.75)	0.99(0.47,2.09)	0.90(0.43,1.89)	1.33(0.61,2.87)	0.69(0.31,1.55)^a^	1.07(0.49,2.34)	0.57(0.27,1.21)^b^	1.73(0.79,3.81)
**Whether the responding patients had previously received community health education service provided by professionals from the the community health center to support chronic disease self-management?**								
•No (Reference)	1.00	1.00	1.00	1.00	1.00	1.00	1.00	1.00
•Yes	1.60(0.76,3.33)^b^	1.64(0.78,3.44)	1.92(0.92,3.99)	0.58(0.27,1.23)	1.74(0.79,3.81)	0.61(0.28,1.31)	2.20(1.04,4.65)	1.34(0.62,2.88)

^a^*p* < 0.05; ^b^*p* < 0.01

### Students’ and faculty members’ attitudes and self-confidence for CSL activities based on the medical student-led community health education service to support chronic disease self-management

The third part of the questionnaire was open to student and faculty respondents to explore their attitudes on the educational benefits of medical student-led community health education service to support chronic disease self-management and their self-confidence in participating in the related CSL activities. As shown in Table 4, both students and faculty members showed their overall positive attitudes toward the educational benefits of the medical student-led community health education service to support chronic disease self-management in promoting the students’ active learning, social skills, professional awareness and career planning. In particular, faculty members appeared to more appreciate the values of this kind of CSL activities in developing professional knowledge and skills, communication skills, problem solving skills, and other social skills (*p* < 0.01).

**Table 4:** Comparison of the students’ and faculty members’ attitudes on the possible educational benefits of the medical student-led community health education service to support chronic disease self-management (Mean ± SD)

Possible educational benefits	Medical students (*n* = 342)	Faculty members (*n* = 54)	*p* value*
•Promoting the students’ active learning to develop professional knowledge and skills.	4.26 ± 0.65	4.52 ± 0.57	0.0055
•Promoting the students’ communication skills, problem solving skills, and other social skills.	4.40 ± 0.63	4.63 ± 0.49	0.0032
•Supporting the medical students’ professional awareness and career planning.	4.24 ± 0.66	4.39 ± 0.56	0.11

* Independent sample *t* test

**Fig. 2:**
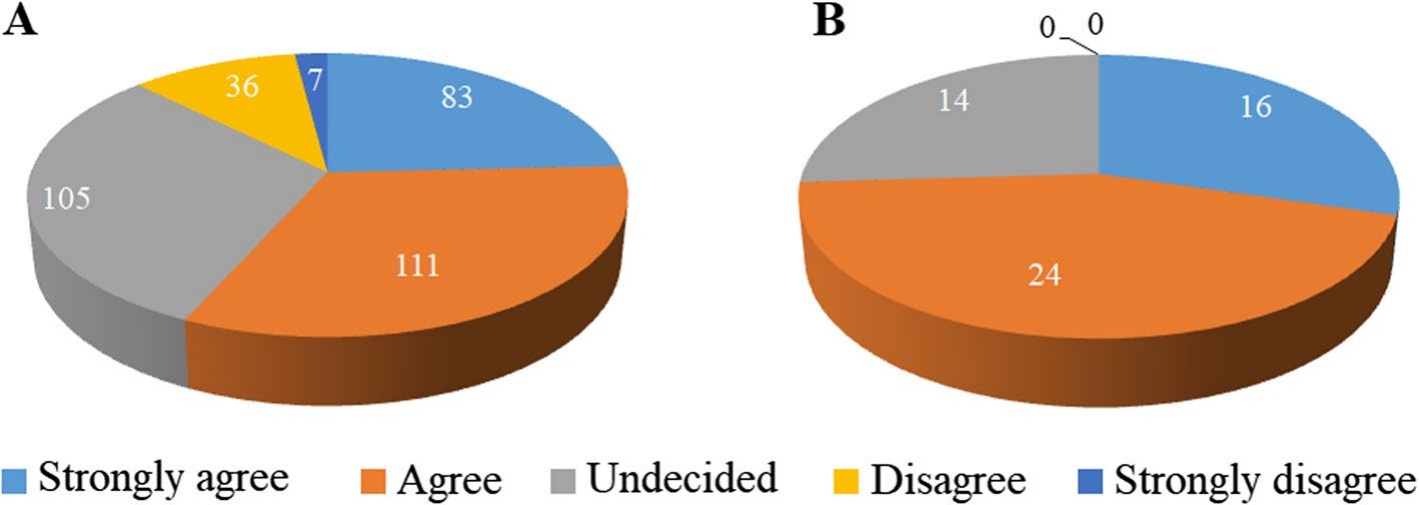
Medical students’ (**A**) and faculty members’ (**B**) attitudes toward the statement of “I am confident in providing (for students)/organizing (for teachers) the CSL programs regarding the medical student-led community service to support chronic disease self-management”

At the end of the questionnaire, the student and faculty respondents were asked about attitudes toward the statement of “I am confident in providing (for students)/organizing (for teachers) the CSL programs regarding the medical student-led community service to support chronic disease self-management”. As shown in Fig. 2, 74.1% of faculty members agreed or strongly agreed and none disagreed this statement, suggesting their self-confidence in organizing the related CSL programs. However, only about half of students (56.7%) agreed or strongly agreed that they were confident in providing the CSL programs regarding the medical student-led community service to support chronic disease self-management.

## Discussion

In the present survey, taking a CSL-based medical student-led community service to support chronic disease self-management as an example, we demonstrated a general uniformity of attitudes toward CSL in medical education among medical students, faculty and patients. The educational value of CSL for medical undergraduates, the interest and availability of medical students in provision of individualized healthcare service were appreciated by most responding students, faculty members and patients; both teachers and medical students showed their agreement with the roles of CSL initiatives based on medical student-led community service to support chronic disease self-management in engaging students in active professional learning, social skill development, and professionalization process. These findings were in line with the known pedagogic advantages of CSL programmes [18, 23–29]. More importantly, some disparities were found to exist among the attitudes of three stakeholder groups, showing that students were more unaware of the current situation of community primary care; patients felt more uncertain of the support of higher medical education system for primary care practice and the students’ competency, and were less interested in participating in the medical student-led community health service; and faculty were more appreciative of CSL and their own roles in CSL programs. The main practical implication of these findings is to provide guidance in encouraging all these key stakeholders to concertedly and effectively participate in the future CSL-based community health service practices for patients with chronic diseases.

Previous studies have demonstrated that the community-engaged learning experience for medical students could produce a win-win situation with patients in the community [31–33]. Cumberland et al. [32] developed a Parkinson’s Disease Buddy Program for medical students, and found that this service learning opportunity not only provided a support system helping students learn about the neurological disease, but also enhanced the Parkinson patients’ self-efficacy and level of socialization that was not originally expected. After participating in a Service-Learning in Communities of Elders, both medical students and elder participants reported enjoyable, valuable experiences obtained from the CSL activities [33]. Being different from the general medical programme focusing on student benefits, CSL places a heavier emphasis on addressing genuine community needs of beneficiaries and encouraging civic engagement [27]. A meaningful CSL activity should promote a teaching-learning reciprocity between the learners and the beneficiaries based on an ethos of partnership [27]. Moreover, the development of modern patient-centred medicine allows patients to act as a more prominent role in medical education and training [28]. Patient contact has long acted as an integral component of learning, teaching and assessment strategies with a positive contribution to medical education [27, 28, 34, 35]. Especially under the modern patient-centred healthcare system, the experiential knowledge of illness shared by patients can expose the medical students to the patient’s perspective, and gain valuable learning experiences and doctor–patient interaction skills, thus can benefit future physicians and patients. In fact, in more and more medical schools, trained patients have served as instructors of clinical skills such as physical examination and communication skills for students as part of the undergraduate medical curricula [27, 28, 34, 35]. For many patients, involvement in the education of tomorrow’s doctors those who will provide healthcare for them is intrinsically an attractive idea [28, 35]. In accord to this notion, the present study found that 63.9% responding patients with chronic disease stated that they were very pleased to participate in the medical student-led community health education service to support chronic disease self-management.

Perhaps because of their own previous visit experiences, patient respondents in this study were more aware of the currently inadequate primary care provision within the community than students. Nevertheless, compared to the students and teachers, patients had lower attitude scores on the CSL-based medical student-led community health education service to support chronic disease self-management. In this study, we found that considerable patient respondents exhibited conservative or hesitant attitudes regarding using resources in higher medical education system to provide support for primary care practice, and participating in the medical student-led community health education service. The concerns might be attributed to their doubts on competency of medical students in supporting chronic disease self-management with their professional knowledge and skills. Given the modest percentage of patients in this cohort who had previously received community health education service provided by professionals to support their chronic disease self-management (51%), it can be speculated that many patients do not know what levels of professional knowledge and skills are required to support the chronic disease self-management, and they had not interacted with medical students thus do not clarify the knowledge and skill levels of students. Patients’ appreciations of CSL activities reported in previous studies [31–33] resulted from their own experience in CSL. Therefore, it is necessary to re-examine the patient respondents’ attitudes toward the medical students’ competency after their personal involvement in the CSL-based medical student-led community health education service.

Moreover, we found that age (≥ 62 years) and number of chronic diseases per patient (> 2) exhibited statistically significant correlation as background factors for patients being more positive toward the CSL-based medical student-led community health education. Despite the fact that the most appropriate target in practice should be the patients who need it the most e.g. those with uncontrolled diabetes mellitus, in view of their positive attitudes and possible higher patient compliance, there is thus the expectation that it might be feasible to first recruit patient volunteers in these population groups to participate in CSL activities based on the medical student-led community health education service to support chronic disease self-management. A previous study identified several patient characteristics influencing the signing of service agreements, i.e. contracts between community patients and primary care physicians [36]. Women, older patients and those with chronic illness were found to be more likely to pay attention to their own health issues and to seek health-related information and service. Consistent with finding from this study [36], the present survey showed more lenient and positive attitudes toward the community health services mainly provided by medical students among the older patients with a great variety of chronic diseases. However, our study showed that patients’ attitudes were not associated with gender.

In addition, faculty plays a central role in service learning projects, yet there is scarce literature about their attitudes and perceptions regarding service-learning [37]. It has been well accepted that having competent faculty instructors to guide and supervise medical students is crucial for CSL to achieve the predefined learning objectives [25, 26, 28, 29, 38]. As opinion leaders who are influential in students’ attitudes, the faculty members play a central important role in shaping medical students’ professional considerations [28]. Furthermore, our attitude survey showed the patients had lower agreement scores in response to the statement ‘Medical students have acquired enough professional knowledge and skills to support chronic disease self-management’, which might be a challenge of medical student-led community service. In this regard, medical educators can play a critical role in addressing this challenge by ensuring students to be well educated about the professional knowledge and skills in this area. Moreover, as for community-academic partnership in CSL, the on-going collaboration with faculty members throughout the project’s lifespan has been mentioned as an opportunity for improvement as well as an integral part in the CSL curriculum from a community partner’s perspective [38]. Encouragingly, in line with the previous survey [29], we found that the faculty respondents were overwhelmingly positive concerning community service activities, and their attitudes were significantly more positive than those of students and patients. Accordingly, a vast majority of faculty members felt self-confident in organizing the CSL programs or activities. In practice, the CSL activities are often designed and applied as case-based teaching and learning approaches, and patient cases in the community context are commonly introduced into classes in medical schools [39, 40]. The medical student-led community health education service to support chronic disease self-management that was studied in this survey has the potential to be introduced into the future case-teaching in the class. Therefore, we explored whether the teachers having experience of case-teaching showed more positive attitudes toward the CSL-based medical student-led community health education service to support chronic disease self-management. However, no difference in case-teaching experience, or each of other sociodemographic features, including gender, profession, or duration of engagement in medical education, was found, suggesting that these variables were not predictive of the faculty attitudes and self-confidence toward CSL.

There are some limitations in this study. This survey only included medical students and faculty members from a single university in China. The patients with chronic diseases were recruited using convenience sampling, and the sample size of patients was not determined by precision analysis technique. The single-center nature and convenience sample methodology of the study might influence the generalisability of findings. Moreover, most attitude questionnaire items in the present study were based on a five-point Likert scale. When comparing the attitudes among students, faculty members and patients, some of the mean attitude scores seemed to be quite close to each other, which indicate a possibility that the statistical significance might not translate to practical differences in the ‘real world’.

## Conclusions

To the best of our knowledge, this is the first study to compare the attitudes toward the medical student-led community health education service to support chronic disease self-management among three stakeholder roles in CSL, including medical students, faculty and patients. The results showed the overall positive attitudes of these stakeholders, more importantly, determined that there were some gaps existing among the attitudes of students, faculty and patients towards the CSL-based medical student-led community health education service to support chronic disease self-management. When comparing attitudes among students, faculty and patients, all three kinds of stakeholders consistently agreed that the medical students are vibrant, enthusiastic, patient and available to provide individualized healthcare service. Faculty members and patients were more worried about the current inadequate level of primary care provision within the community. Patients’ enthusiasm about CSL based on medical student-led community health education service to support chronic disease self-management appeared to be below the expectations of students and teachers. Patient respondents were also most skeptical about the competency of medical students in supporting chronic disease self-management with their professional knowledge and skills. Relatively, faculty members most appreciated the educational benefits of CSL for medical undergraduates and the role of faculty instructors. Most patients wanted to participate in the online community health education service led by medical students. Moreover, our results showed some background factors that correlated to the positive patients’ attitudes, including being more than 62 years old, and having more than 2 kinds of chronic diseases, suggesting those were the most amenable patient groups.

Based on the above findings, in order to better support the future CSL activities in medical education as well as the community health service practices for patients with chronic diseases, we concluded some recommendations as follow:

1. Allowing medical students to better understand the current role of community-based primary care in chronic disease management.
2. Strengthening students’ professional knowledge, skills and their self-confidence in supporting chronic disease self-management to develop their competency in community health education service.
3. Further promoting the training of faculty instructors who are competent to guide and supervise medical students in CSL programs based on the medical student-led community health education service to support chronic disease self-management.
4. Recruiting patient volunteers from the aged individuals with a great variety of chronic diseases first, based on their positive attitudes and possible higher patient compliance.
5. The online mode might be quite appropriate for the medical student-led community health education service to support chronic disease self-management especially after the outbreak of COVID-19.

## Data Availability

The datasets used and/or analysed during the current study are available from the corresponding author on reasonable request.

## Abbreviations

ANOVA: One-way Analysis of Variance
COVID-19: Coronavirus Disease 2019
CSL: Community Service Learning
SD: Standard Deviation
